# Metformin Potentiates the Anticancer Effect of Everolimus on Cervical Cancer In Vitro and In Vivo

**DOI:** 10.3390/cancers13184612

**Published:** 2021-09-14

**Authors:** Ya-Hui Chen, Jyun-Xue Wu, Shun-Fa Yang, Mei-Ling Chen, Tze-Ho Chen, Yi-Hsuan Hsiao

**Affiliations:** 1 Women’s Health Research Laboratory, Changhua Christian Hospital, Changhua 50006, Taiwan; 106317@cch.org.tw (Y.-H.C.); 183726@cch.org.tw (J.-X.W.); 2 Institute of Medicine, Chung Shan Medical University, Taichung 40201, Taiwan; ysf@csmu.edu.tw; 3 Department of Medical Research, Chung Shan Medical University Hospital, Taichung 40201, Taiwan; 4 Department of Pathology, Changhua Christian Hospital, Changhua 50006, Taiwan; 55828@cch.org.tw; 5 Department of Obstetrics and Gynecology, Changhua Christian Hospital, Changhua 50006, Taiwan; 46305@cch.org.tw; 6 School of Medicine, Chung Shan Medical University, Taichung 40201, Taiwan; 7 College of Medicine, Kaohsiung Medical University, Kaohsiung 807378, Taiwan

**Keywords:** cervical cancer, metformin, everolimus, apoptosis, mtROS, PI3K/AKT, p38/ERK/JNK

## Abstract

**Simple Summary:**

Recent studies have shown that metformin combined with clinical chemotherapeutic drugs could cause decreased cell toxicity and attenuate tumor resistance in various types of cancer. The aim of the present study was to elucidate whether combined treatment with metformin and everolimus has a synergistic anticancer effect in human cervical cancer in vitro and in vivo. The results showed that this combined treatment synergistically inhibited the growth of human cervical cancer cell lines and xenografts in nude mice, and induced caspase-dependent apoptosis, promoting sub-G1- and G0/G1-phase arrest and enhancing mtROS production. Combined treatment also synergistically inactivated PI3K/AKT signaling and activated MAPKs signaling in cervical cancer. Our data suggested that metformin potentiates the anticancer effect of everolimus on cervical cancer, and combined treatment provides a novel therapeutic strategy for patients with cervical cancer.

**Abstract:**

Cervical cancer is globally the fourth most common cancer in women. Metformin is a widely used drug for the treatment of type II diabetes and has been shown to possess important anticancer properties in cervical cancer. Everolimus is an mTOR inhibitor and is widely used to treat NETs, RCC, TSC, and breast cancers. The present study investigated the anticancer effects of metformin and everolimus in cervical cancer, when used alone or in combination. CaSki and C33A human cervical cancer cells were treated with different concentrations of everolimus alone or in combination with metformin. Cell viability was assessed using a CCK-8 assay. Cell apoptosis, cell-cycle, and mtROS analyses were conducted using flow cytometry. Target protein levels were analyzed by Western blotting. Related mechanisms were confirmed using appropriate inhibitors (z-VAD-fmk and BIRB796). The in vitro results were further confirmed in a xenograft tumor study. Both metformin and everolimus, when used alone, were moderately effective in inhibiting cell proliferation and inducing cell apoptosis of CaSki and C33A cells. When used in combination, these two drugs synergistically inhibited the growth of human cervical cancer cells and xenografts in nude mice, promoted sub-G1- and G0/G1-phase cell-cycle arrest, and enhanced mtROS production. The protein expressions of PI3K (p110α) and p-AKT were significantly downregulated, while P27, P21, p-p38, p-ERK, and p-JNK were upregulated following combined treatment. These results revealed that metformin potentiates the anticancer effect of everolimus on cervical cancer, and combination treatment with metformin and everolimus provides a novel therapeutic strategy for patients with cervical cancer.

## 1. Introduction

Cervical cancer is globally a major gynecologic carcinoma among women; in 2018, approximately 570,000 women were diagnosed and more than 310,000 women died from the disease [1]. Nowadays, although there have been significant advances in surgery, chemotherapy, and radiotherapy, there still exist many serious problems, such as recurrence, metastasis, severe side effects, and drug resistance; the overall 5-year survival rate is 66.3% according to SEER18 2011–2017 [2]. The Global Cancer Observatory reported that the global burden of cervical cancer continues to increase and is projected to rise to 700,000 cases and 400,000 deaths by 2030 [3]. In studies regarding international trends in the incidence of cervical cancer based on pathological type, squamous cell carcinoma represents three-quarters of cases; adenosquamous cell carcinoma and adenocarcinoma account for 10–15%; and other types or those of unspecified histology account for the remaining 10–15% of cases [4,5]. Furthermore, histology has been shown to be a statistically significant variable in cause-specific mortality [6]. Relative to non-microinvasive squamous cell carcinoma, the cause-specific mortality hazard ratios for microinvasive squamous cell carcinoma and adenosquamous carcinoma were reported to be 0.28 (95% confidence interval (CI: 0.20–0.39) and 1.35 (95% CI: 1.20–1.51), respectively; while the hazard ratio for non-mucinous adenocarcinoma was 1.06 (95% CI: 0.98–1.15) and that of mucinous adenocarcinoma was 1.52 (95% CI: 1.23–1.88) [6]. Thus, novel treatments to inhibit tumor growth and improve the survival rate of cervical cancer patients need to be developed.

Metformin (N-dimethylniguanide) is a widely used lipophilic biguanide drug for the treatment of type II diabetes in the early stages. Numerous studies have shown that metformin could reduce the risk and increase the survival of diabetic patients with hepatocellular carcinoma (HCC) [7], breast cancer [8], and lung cancer [9]. Moreover, Hanprasertpong et al. showed that the use of metformin in cervical cancer patients with diabetes could result in a lower recurrence rate and improve disease-free survival as compared with patients who do not receive metformin [10]. Takiuchi et al., however, revealed no association between metformin use and survival outcome in women with cervical cancer [11]. Several studies have also shown that metformin could inhibit the cellular proliferation and tumor growth of various cancer cells both in vitro and in vivo, such as breast, lymphoma, endometrial, HCC, and thyroid cancer, and metformin exerts its anticancer effects through the human epidermal growth factor receptor-2 (HER-2), AMP-activated protein kinase (AMPK), mammalian target of rapamycin (mTOR), insulin-like growth factor-1 (IGF-1), c-Jun N-terminal kinase/mitogen-activated protein kinase (JNK/p38 MAPK), and nuclear factor kappaB (NF-κB) pathways [12,13,14,15,16,17].

Earlier clinical studies indicated that metformin could improve chemotherapy outcomes, reduce the mortality rate, and increase the overall survival rate of patients with breast cancer (NCT00930579, NCT00984490) [18], prostate cancer (NCT00881725) [18], non-small cell lung cancer [19,20], and head and neck cancer [21] by activating AMPK, inhibiting PI3K/AKT/mTOR, and inactivating MAPK. A recent phase II clinical study showed that metformin improved the fractional hypoxic volume of a tumor on a FAZA-PET scan in locally advanced cervix cancer (LCAA, NCT02394652), and was also expected to alter tumor oxygenation to overcome radiotherapy resistance in LCAA (NCT04275713). Therefore, recent in vitro and in vivo studies indicated that metformin combined with caffeic acid or nelfinavir synergistically inhibited cervical cancer cell growth and tumor growth [22,23]. Table 1 presents details of several clinical trials related to metformin combined with other agents (e.g., megestrol acetate, irinotecan, gemcitabine, erlotinib, (Ir)relevance, nivolumab, and ipilimumab), performed to identify whether these treatments can improve the survival of patients with endometrial, colon, pancreatic, ovarian, prostate, or breast cancer [24,25,26,27,28,29,30]. Thus, understanding the underlying mechanism of metformin is critical to developing potential combination therapies for cervical cancer.

Everolimus (RAD001) is a rapamycin analog with a similar function to rapamycin, as a protein kinase inhibitor of the mTOR serine/threonine kinase signal transduction pathway. Everolimus has been approved for the treatment of pancreatic neuroendocrine tumors (p-NETs), advanced renal cell carcinoma (RCC), subependymal giant cell astrocytoma (SEGA) associated with tuberous sclerosis complex (TSC), and in combination with exemestane for advanced hormone-receptor (HR)-positive, HER2-negative breast cancer [31,32]. In 2019, Taylor et al. discovered in a phase I/II study of everolimus and bevacizumab in advanced solid tumors (e.g., ovarian, peritoneal, and fallopian tube cancers) that this proved to be a promising combination treatment [33]. In addition, in vitro and in vivo studies indicated that everolimus combined with metformin synergistically inhibited breast cancer cell growth, clonogenicity, PI3K/mTOR signaling activity, mitochondrial respiration, and xenograft tumor growth [34,35]. There are also several ongoing phase II clinical trials related to metformin combined with everolimus in patients with advanced pancreatic neuroendocrine tumors, endometrial cancer, and breast cancer [36,37]. In addition, our recent studies showed that metformin induces cell apoptosis and cell-cycle arrest, and inhibits cellular proliferation in cervical cancer cells [38]. Moreover, metformin combined with pitavastatin enhances the anticancer effects to induce cell apoptosis and autophagy in pancreatic cancer cells [39]. Therefore, in the present study, we investigated the growth-inhibitory effects and underlying molecular mechanisms of metformin and everolimus treatment alone and in combination on cervical cancer cell lines.

## 2. Materials and Methods

### 2.1. Cell Lines and Culture Conditions

CaSki and C33A human cervical cancer cell lines were purchased from the Bioresource Collection and Research Center (BCRC, Hsinchu, Taiwan; derived from ATCC CRM-1550TM and ATCC CRM-HTB-31). CaSki cells were cultured in an RPMI 1640 medium (1-41P05-K, BioConcept, Amimed, Allschwil, Switzerland), and C33A cells in Eagle’s Minimum Essential Medium (SH30024.02, MEM, Cytiva, Marlborough, MA, USA) supplemented with 10% fetal bovine serum (SH30396.03, FBS, Cytiva) at 37 °C in a humidified incubator with 5% CO_2_.

### 2.2. CCK-8 Assay and Analysis of Drug Synergy

Cell viability was measured using a Cell Counting Kit-8 assay (#CK04, CCK-8, Dojindo Molecular Technologies, Inc., Rockville, MD, USA). In light of previous studies indicating that cancer cell treatment with 5~20 mM metformin or 15~25 μM everolimus effectively reduced cell proliferation [40,41,42], CaSki and C33A cells (2 × 10^4^/well) were seeded into 96-well plates and then treated with different concentrations (10 and 20 mM in sterilized water) of metformin (#D150959, Sigma-Aldrich, St. Louis, MO, USA) with or without (10 and 20 μM in dimethyl sulfoxide, Sigma-Aldrich) everolimus (#S1120, Selleck Chemicals, Houston, TX, USA) for 48 h. Control cells were treated with 0.1% DMSO in the culture medium. Subsequently, CCK-8 (10 μL) was added to each well, and cells were incubated for 1 h at 37 °C. Absorbance at 450 nm was measured using a microplate reader (FLUOstar Galaxy, BMG Labtech, Ortenberg, Germany). The synergistic effect of the drugs was analyzed using the Chou–Talalay method and CalcuSyn software (Biosoft, Cambridge, UK) [43]. A combination index (CI) value of <1 indicated synergy, >1 indicated antagonism, and 1 indicated an additive effect of the two agents. In further experiments, cells were pretreated with various inhibitors, such as z-VAD-fmk (#1009-20C, 10 μM, BioVision, Inc., Milpitas, CA, USA) and BIRB796 (#S1574, 5 μM, Selleck Chemicals), prior to metformin and everolimus treatment.

### 2.3. Cell-Cycle and Apoptosis Analysis

CaSki and C33A cells were seeded into 6-well plates at a density of 1 × 10^6^ cells per well and exposed to 10 mM metformin with or without 20 μM everolimus for 48 h. Cells were then collected and fixed with 70% ice-cold ethanol at −20 °C overnight. After fixation, cells were centrifuged at 400× *g* for 10 min at 4 °C, washed with cold PBS, and stained with 0.5 mL PI/RNases staining buffer (PI, 10 μg/mL; RNases, 300 μg/mL; #550825, BD Pharmingen; BD Biosciences, Franklin Lakes, NJ, USA) for 15 min at room temperature in the dark. Cell apoptosis was detected using an FITC Annexin V apoptosis detection kit (#556547, BD Biosciences); cells were double-stained with 5 μL FITC Annexin V (20 μg/mL) and 5 μL propidium iodide (PI, 50 μg/mL) for 15 min at RT in the dark. Finally, the stained cells were analyzed using FC500 flow cytometry and CXP software (version 2.3; Beckman Coulter, Brea, CA, USA), and early and late apoptotic or necrotic cells were assessed.

### 2.4. Mitochondrial ROS Measurement

For the measurement of mitochondrial reactive oxygen species (ROS) generation, cells were assessed using the MitoSOX™ Red mitochondrial superoxide indicator (#M36008, Invitrogen, Thermo Fisher Scientific, Waltham, MA, USA) according to the manufacturer’s instructions. CaSki and C33A cells were plated in 6-well plates with a cell density of 1 × 10^6^ cells/well and treated with 10 mM metformin with or without 20 μM everolimus for 48 h. After treatment, cells were stained with 2.5 μM of MitoSOX Red reagent, shaken, placed in a 37 °C water bath, and incubated in the dark for 20 min. After incubation, fluorescence signals were detected using a flow cytometer (Cytomics FC 500, Beckman Coulter) and FlowJo software (version 7.6; BD Life Sciences, Franklin Lakes, NJ, USA), and are represented as percentages of the results of the control group.

### 2.5. Western Blot Analysis

CaSki and C33A cells were seeded (2 × 10^6^ cells/dish) into 10 cm dishes, pretreated with or without z-VAD-fmk (10 μM) and BIRB796 (5 μM) for 2 h at 37 °C, and incubated with 10 mM metformin with or without 20 μM everolimus for 48 h. Cells were lysed in RIPA buffer (#20-188, Millipore, Billerica, MA, USA), and protein concentrations were measured using a BCA protein assay kit (#23225, Thermo Fisher Scientific). Proteins (30 µg) were incubated at 95 °C for 10 min, separated on 10–12% (*w*/*v*) SDS-PAGE, and transferred onto 0.2-µm PVDF membranes (Bio-Rad, Irvine, CA, USA). Following blocking with a BlockPRO blocking buffer (Energenesis Biomedical) for 1 h at room temperature, membranes were probed with the following primary antibodies (1:1000 dilution) at 4 °C overnight: PARP (#9532), Bcl-2 (#15071), Bax (#5023), cleaved caspase-3 (#9664), P27Kip1 (#3686), phospho-AKT (Ser473) (#4060), Akt (#4298), phospho-P38 (#9211), P38 (#9212), phospho-ERK1/2 (#4370), ERK1/2 (#4695), phospho-JNK (#4668), JNK (#9258; Cell Signaling, Danvers, MA, USA); P21Cip1 (GTX629543), PCNA (GTX100539; GeneTex, Inc., Irvine, CA, USA); PIK3CA (NBP2-19804; Novus, CO, USA); MitoProfile^®^ Total OXPHOS rodent antibody cocktail (ab110413; Abcam, Cambridge, UK) and GAPDH (AC002, ABclonal Biotech, Woburn, MA, USA). Then, the membranes were washed and incubated with a secondary IgG-HRP-conjugated antibody (Mouse#115-035-003; Rabbit#111-035-003; Jackson ImmunoResearch, Laboratories, Inc., West Grove, PA, USA) for 1 h at room temperature. Protein bands were obtained using an enhanced chemiluminescence reagent (#WBKLS0500, ECL, Millipore, Burlington, MA, USA), and densitometry was performed using Fusion-Capt Advanced FX7 software (version 16.08a; Labtech International, Inc., Collégien, France).

### 2.6. C33A Tumor Xenograft Model

Seven-week-old BALB/c nude female mice were purchased from the National Laboratory Animal Center (Taipei, Taiwan). The mice were housed under standard conditions (12:12 h light/dark cycle at 22 °C) in specific pathogen-free conditions with ad libitum access to water and food and maintained under the care of facility staff according to the guidelines of the animal ethics committee. All animal experimental protocols and facilities were reviewed and approved by the Institutional Animal Care and Use Committee (IACUC) of Changhua Christian Hospital, Taiwan (approval no: CCH-AE-110-001).

All cancer cell implantations were performed under 2–3% isoflurane (Panion & BF Biotech) inhalation, and all efforts were made to minimize suffering. Human cervical cancer C33A cells were mixed with Corning^®^Matrigel^®^ Matrix reagent (#354248, Corning, Tewksbury, MA, USA) at a 2:1 ratio, and each BALB/c nude mouse was subcutaneously injected in the right flank with 1 × 10^7^ cells. Nine days later, the tumor volume reached ~200 mm^3^, and the mice were randomly divided into four groups (four mice per group): vehicle control group (10% DMSO, 40% Cremophor/ethanol (3:1; #C5135, Sigma-Aldrich, St. Louis, MO, USA) and 50% PBS); metformin group (150 mg/kg/day, dissolved in 10% sterilized water, 40% Cremophor/ethanol (3:1) and 50% PBS, intraperitoneally); everolimus group (5 mg/kg/day, dissolved in 10% DMSO, 40% Cremophor/ethanol and 50% PBS, intraperitoneally); and combination group (metformin 150 mg/kg/day plus everolimus 5 mg/kg/day, intraperitoneally). Tumor volume and mouse body weight were measured using an electronic caliper every two days after the initial injection. Tumor volume was calculated using the following standard formula: volume = length × width^2^/2. After 16 days, the mice were sacrificed using a CO_2_ chamber, and the tumors were extracted for tissue analysis.

### 2.7. Histopathology and Immunohistochemistry

Fresh tumor tissue was formaldehyde (10%)-fixed and paraffin-embedded, and 5 μM sections were cut. Primary tumors were stained with H&E for histological examination and morphometric analysis. For immunohistochemical staining, slides were deparaffinized and rehydrated, and antigens were retrieved in boiling ddH_2_O for 10 min. Slides were then incubated in 3% H_2_O_2_ for 10 min to inhibit endogenous peroxidase activity and stained with antibodies against Ki67 (#12202; 1:400), cleaved caspase-3 (#9664; 1:100), and phospho-P38 (#9211; 1:200, Cell Signaling) for 3 h at 37 °C. Slides were then washed and incubated with OneStep Polymer HRP-conjugated anti-mouse/rat/rabbit IgG secondary antibody (GTX83398; GeneTex) for 30 min at room temperature, then visualized using a colorimetric method (DAB kit; GTX30939; GeneTex). Nuclei were counterstained with hematoxylin and photographed using an Olympus BX61 microscope (Tokyo, Japan). With regards to animal tumor tissue, positive cells were quantified in three fields per four mice for each group (Image-Pro Plus 4.5).

### 2.8. Statistical Analysis

All numerical data are presented as the mean ± standard deviation (SD), and all data represent results from at least three independent experiments. All analyses were performed using the Student’s *t*-test. *p* < 0.05 was considered significant.

## 3. Results

### 3.1. Combined Treatment with Metformin and Everolimus Is More Effective than Either Treatment Alone in Inhibiting Cell Viability

**Figure 1:**
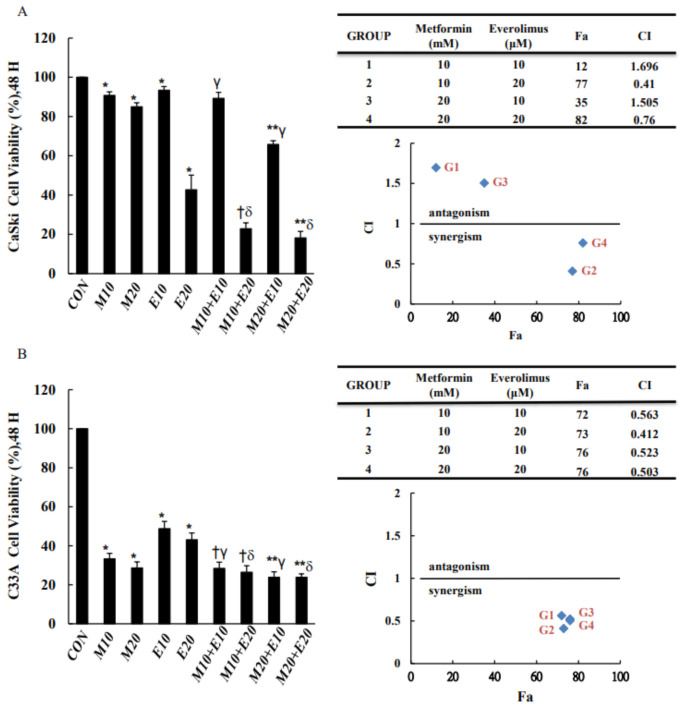
Metformin and everolimus inhibited CaSki and C33A cell growth. Inhibition effects of metformin (10, 20 mM) or everolimus (10, 20 μM) on (**A**) CaSki and (**B**) C33A cell growth measured using a CCK-8 assay. Cells were exposed to metformin with or without everolimus for 48 h, then subjected to CCK-8 assay. CIs of metformin combined with everolimus were calculated using Chou–Talay analyses. Values represent the mean ± SD from three replicates. *, †, γ, ** and δ *p* < 0.05 compared with CON, Met 10- or Eve 10- or Met 20- or Eve 20-treated groups. CON, 0.1% DMSO; M10, 10 mM metformin; M20, 20 mM metformin; E10, 10 μM everolimus; E20, 20 μM everolimus. CI, combination index; Fa, affected fraction.

To assess the effects of both metformin and everolimus on the viability of human CaSki and C33A cells, cells were treated with various concentrations of metformin (10, 20 mM) with or without everolimus (10, 20 μM) for 48 h, and assayed for viability using the CCK-8 method. The results of the dose–response studies are shown in Figure 1. CaSki and C33A cervical cancer cell growth was decreased by both metformin and everolimus; the antiproliferative effect of combined metformin and everolimus on cervical cancer cells was significantly greater than that of metformin or everolimus alone, particularly 20 μM everolimus combined with 10 or 20 mM metformin (approximately 74–82% inhibition efficiency, *p* < 0.05). Similar results were obtained in that metformin combined with everolimus synergistically inhibited cell viability, with a CI of 0.41 and 0.76 for 10 and 20 mM metformin with 20 μM everolimus for CaSki cells, and 0.412 and 0.503 for 10 and 20 mM metformin with 20 μM everolimus for C33A cells. Together, these results suggested that metformin combined with everolimus could have a synergistic effect against the cell proliferation of cervical cancer cells. In subsequent experiments, the anticancer effect of 10 mM metformin combined with 20 μM everolimus on cervical cancer cells was assessed; these concentrations were selected based on the CI assay, which indicated that they resulted in the greatest synergistic effect as compared with the other combined treatment groups.

### 3.2. Combined Treatment with Metformin and Everolimus Is More Effective than Either Treatment Alone in Promoting Cell Apoptosis and Cell-Cycle Arrest

**Figure 2:**
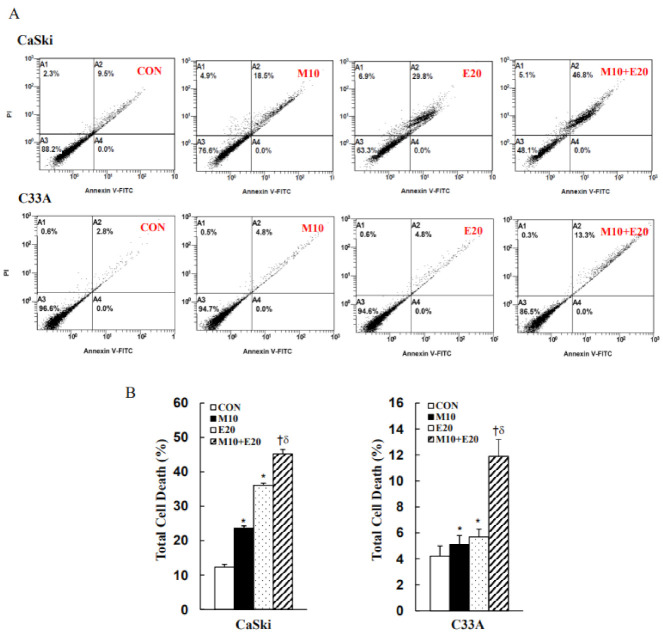
Metformin and everolimus induced apoptosis in cervical cancer cells. CaSki and C33A cells were treated with metformin (10 mM) with or without everolimus (20 μM) for 48 h. (**A**) Apoptosis profile assessed using an Annexin V/PI assay by flow cytometry. (**B**) Quantitative results of apoptotic cells (Annexin V- and PI-stained cells) presented in the bottom plot. Values represent the mean ± SD from three replicates. *, † and δ *p* < 0.05 compared with CON, Met 10- or Eve 20-treated groups. CON, 0.1% DMSO; M10, 10 mM metformin; E20, 20 μM everolimus.

**Figure 3:**
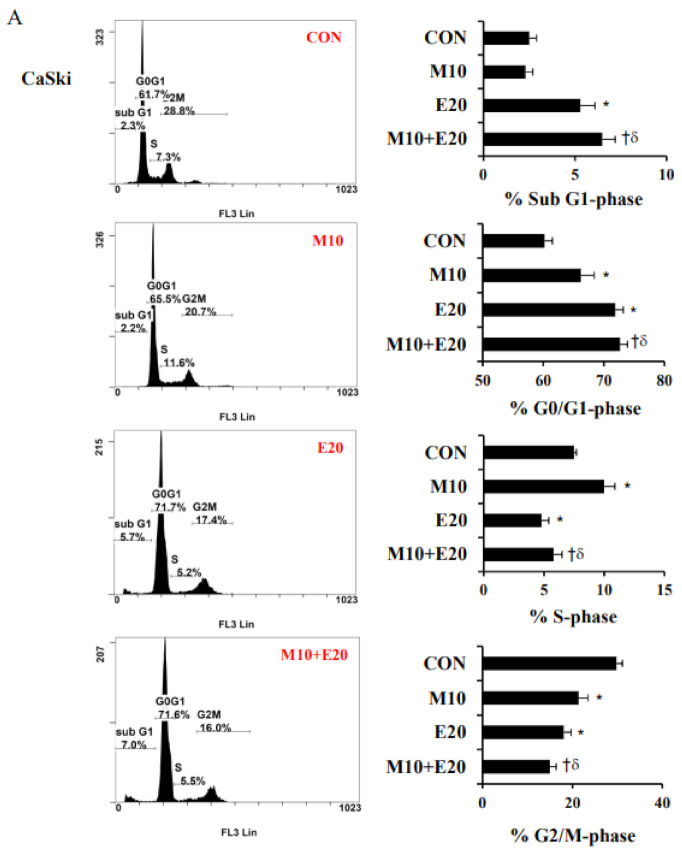

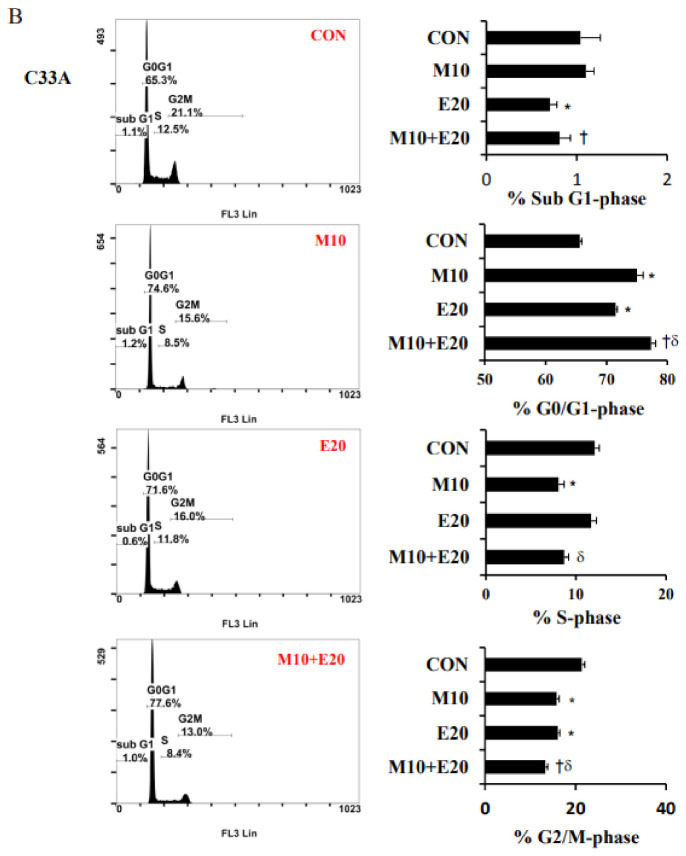
Metformin and everolimus arrested the cell cycle in the sub-G1 and G0/G1 phases. (**A**) CaSki and (**B**) C33A cells were treated with metformin (10 mM) with or without everolimus (20 μM) for 48 h. Cell-cycle progression was assessed using a PI assay via flow cytometry. Quantitative results for the cell-cycle phase distribution (sub-G1, G0/G1, S, and G2/M phases) are presented in the bottom plot. Values represent the mean ± SD from three replicates. *, † and δ *p* < 0.05 compared with the CON, Met 10- or Eve 20-treated groups. CON, 0.1% DMSO; M10, 10 mM metformin; E20, 20 μM everolimus.

To determine whether the apoptosis and cell-cycle processes were involved in the inhibition of cervical cancer cell proliferation by treatment with metformin and/or everolimus, CaSki and C33A cells were stained with Annexin V/PI or PI/RNases and analyzed via flow cytometry. As shown in Figure 2, the numbers of cells in the late apoptotic or necrotic phases (PI+/annexin V+) and early apoptotic phases (PI–/annexin V+) were significantly increased following treatment with metformin and everolimus (23.6 ± 3.6% for 10 mM metformin, 36.1 ± 3.5% for 20 μM everolimus in the case of CaSki cells; 5.1 ± 0.7% for 10 mM metformin, 5.7 ± 0.6% for 20 μM everolimus in the case of C33A cells) as compared with the control (12.3 ± 2.3% for CaSki cells; 4.2 ± 0.8% for C33A cells). As expected, combined treatment with metformin (10 mM) and everolimus (20 μM) significantly increased the numbers of cells in the late apoptotic or necrotic phases and the early apoptotic phase (45.2 ± 7.9% for CaSki cells; 11.9 ± 1.3% for C33A cells) as compared with metformin or everolimus alone, with increases of 1.3- to 2.3-fold (*p* < 0.05). In a cell-cycle distribution assay, metformin or everolimus treatment resulted in increased sub-G1-phase cells from 2.5 ± 0.4% (untreated cells) to 5.3 ± 0.8% (20 μM everolimus), and significantly increased G0/G1-phase cells from 60.2 ± 1.3% (untreated cells) to 66.2 ± 2.2% (10 mM metformin) and to 71.9 ± 1.3% (20 μM everolimus) in the case of CaSki cells. Simultaneously, the percentage of G0/G1-phase cells increased from 65.6 ± 0.3% (untreated cells) to 75.0 ± 1.0% (10 mM metformin) and to 71.5 ± 0.3% (20 μM everolimus) in the case of C33A cells. Combined treatment with metformin and everolimus resulted in significantly higher percentages of sub-G1- and G0/G1-phase cells (6.5 ± 0.7% vs. 72.7 ± 1.2% in the 10 mM metformin +20 μM everolimus group) in CaSki cells, and only the percentage of cells in the G0/G1-phase was significantly higher (77.3 ± 0.8%) in C33A cells, as compared with cells treated with metformin or everolimus alone (Figure 3, *p* < 0.05). Together, these results suggested that combined treatment with metformin and everolimus was more effective than treatment with either alone in promoting human cervical cancer cell apoptosis and causing cell-cycle arrest in the sub-G1 and G0/G1 phases.

### 3.3. Combined Treatment with Metformin and Everolimus Synergistically Enhances Mitochondrial ROS and OXPHOS

**Figure 4:**
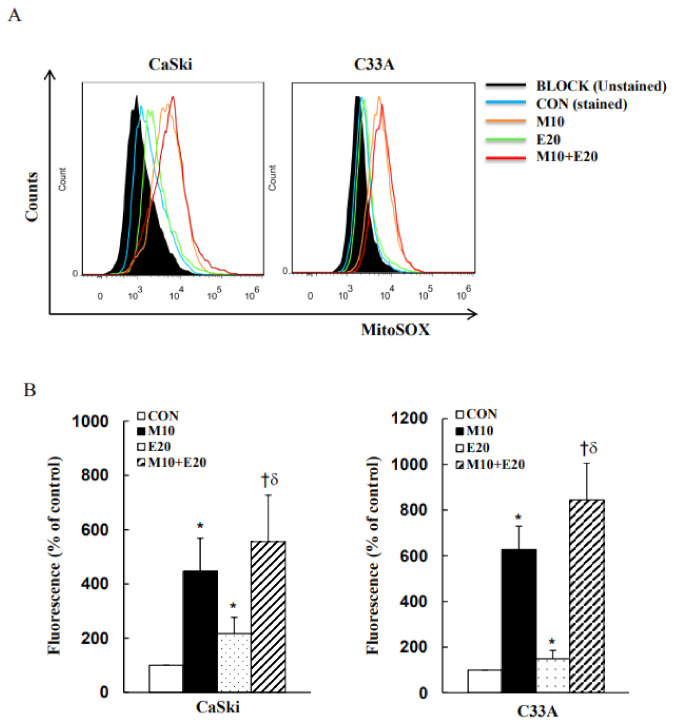

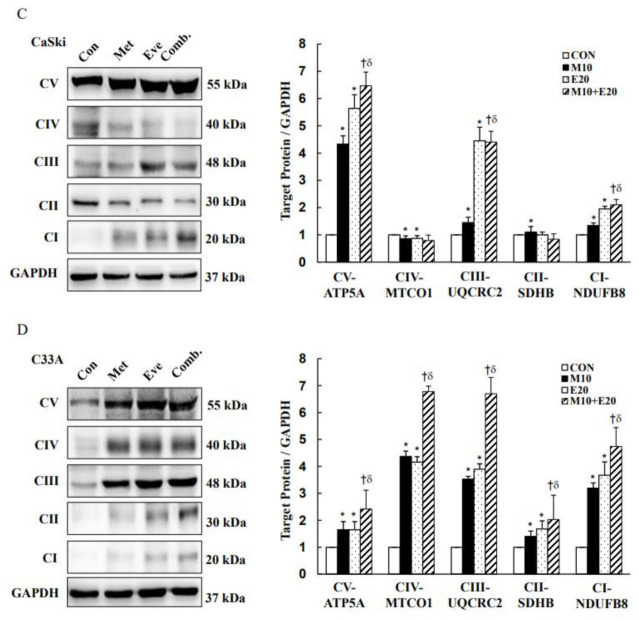
Metformin and everolimus enhanced mitoROS-mediated OXPHOS expression in cervical cancer cells. CaSki and C33A cells were treated with metformin (10 mM) with or without everolimus (20 μM) for 48 h. (**A**) Mitochondrial ROS levels measured using MitoSOX Red reagent via flow cytometry. (**B**) Quantitative results of mitochondrial ROS levels presented in the bottom plot. (**C**,**D**) Protein expression levels of OXPHOS (complex I–V) measured using Western blotting. Quantitative results presented in the bottom plot. Values represent the mean ± SD from three replicates. *, † and δ *p* < 0.05 compared with CON, Met 10- or Eve 20-treated groups. CON, 0.1% DMSO; M10, 10 mM metformin; E20, 20 μM everolimus.

Mitochondria are indispensable regulators of apoptosis, redox balance, and cell signaling. Mitochondrial functions are usually altered in malignant cells, including resistance to apoptosis, oxidative phosphorylation (OXPHOS) deficit, and mitochondrial ROS overproduction. To better understand the role of mitochondria in cervical cancer development, mitochondrial ROS and OXPHOS levels were examined using the MitoSOX Red reagent and Western blot analysis. MitoSOX Red-derived fluorescence levels were significantly increased following treatment with 10 mM metformin and 20 μM everolimus in CaSki and C33A cells as compared with the control (non-treated) cells (Figure 4A,B; *p* < 0.05). Combined treatment with metformin and everolimus further increased the MitoSOX Red-derived fluorescence levels of CaSki and C33A cells as compared with treatment with metformin or everolimus alone. In addition, CaSki cells showed increases in the expressions of Complex V, III, and I proteins (3.33-, 0.45-, and 0.34-fold, respectively) and a reduction in the expression of Complex IV (−0.14-fold) when treated with 10 mM metformin alone, while Complex II did not change; moreover, when CaSki cells were treated with 20 μM everolimus alone, 4.64-, 3.45-, and 0.95-fold upregulations were noted in the expressions of Complex V, III, and I proteins, and a −0.13-fold downregulation in the expression of Complex IV was observed as compared with the non-treated control cells (Figure 4C and Figure S1A, *p* < 0.05); Complex II was unchanged. In the case of C33A cells, the expressions of Complex V (0.65- and 0.65-fold, respectively), IV (3.37- and 3.16-fold, respectively), III (2.53- and 2.9-fold, respectively), II (0.4- and 0.68-fold, respectively), and I (2.19- and 2.67-fold, respectively) proteins were significantly altered upon treatment with 10 mM metformin or 20 μM everolimus alone as compared with the non-treated control cells (Figure 4D and Figure S1B, *p* < 0.05). Compared with metformin or everolimus treatment alone, combination treatment resulted in significantly higher expressions of Complex I–V proteins in CaSki and C33A cells, although Complex IV and II proteins in CaSki cells were not increased, suggesting that metformin and everolimus treatment could improve mitochondrial respiration in human cervical cancer CaSki and C33A cells. Our results indicated that metformin combined with everolimus induced cancer cell apoptosis or necrosis in association with enhanced mitochondrial ROS, and increased the mitochondrial OXPHOS level to preserve mitochondrial function.

### 3.4. Combined Treatment with Metformin and Everolimus Activates Mitochondrial and Caspase-Mediated Apoptosis

**Figure 5:**
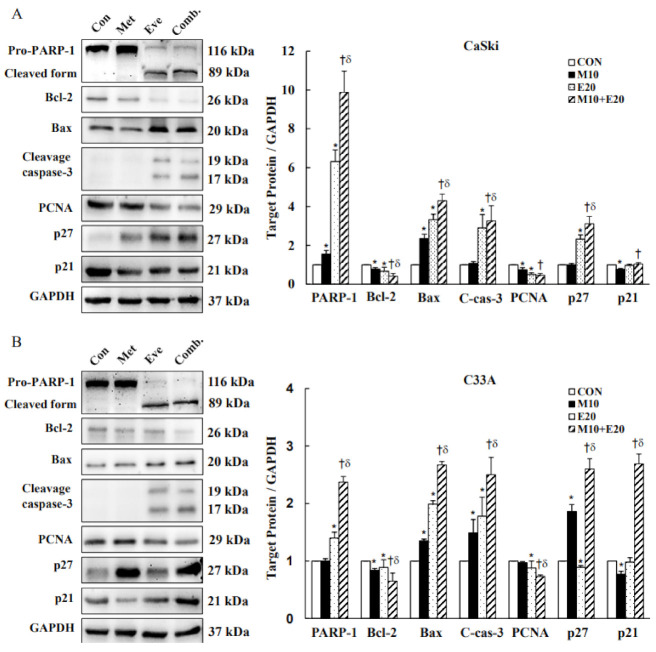
Metformin and everolimus induced apoptosis through mitochondrial and caspase-mediated pathways. Representative Western blot data showing expressions of apoptosis-related proteins in (**A**) CaSki and (**B**) C33A cells treated with metformin (10 mM) with or without everolimus (20 μM) for 48 h. GAPDH served as the loading control. Quantitative results are presented in the bottom plot. Values represent the mean ± SD from three replicates. *, † and δ *p* < 0.05 compared with the CON, Met 10- or Eve 20-treated groups. CON, 0.1% DMSO; M10, 10 mM metformin; E20, 20 μM everolimus.

To confirm our prior results that metformin and everolimus treatment induced cancer cell death by apoptosis and cell-cycle arrest, we determined the expressions of apoptosis-, replication-, and cell-cycle-related proteins, including PARP-1, Bcl-2, Bax, caspase-3, PCNA, P27, and P21, by Western blotting. We found that 10 mM metformin and 20 μM everolimus monotherapy significantly downregulated antiapoptotic protein Bcl-2 and proliferation marker PCNA expressions, and upregulated proapoptotic protein Bax, cleaved-caspase-3, and cleaved-PARP-1 protein expression levels as compared with the control in cervical cancer CaSki and C33A cells. Metformin with everolimus combination treatment more significantly downregulated Bcl-2 and PCNA expressions, and upregulated Bax, cleaved-caspase-3, and cleaved-PARP-1 expressions as compared with metformin or everolimus monotherapy. Moreover, metformin with everolimus combination treatment significantly increased P27Kip1 and P21Cip expression levels as compared with metformin or everolimus monotherapy (Figure 5 and Figure S2, *p* < 0.05). Thus, these data showed that the metformin- and everolimus-induced apoptosis of CaSki and C33A cells occurred through a mitochondria-mediated pathway, and activated caspase-3 and PARP-1 were involved in the apoptotic effect.

### 3.5. Combined Treatment with Metformin and Everolimus Synergistically Inhibits PI3K(p110α)/AKT Signaling and Activates MAPKs Signaling in Cervical Cancer Cells

**Figure 6:**
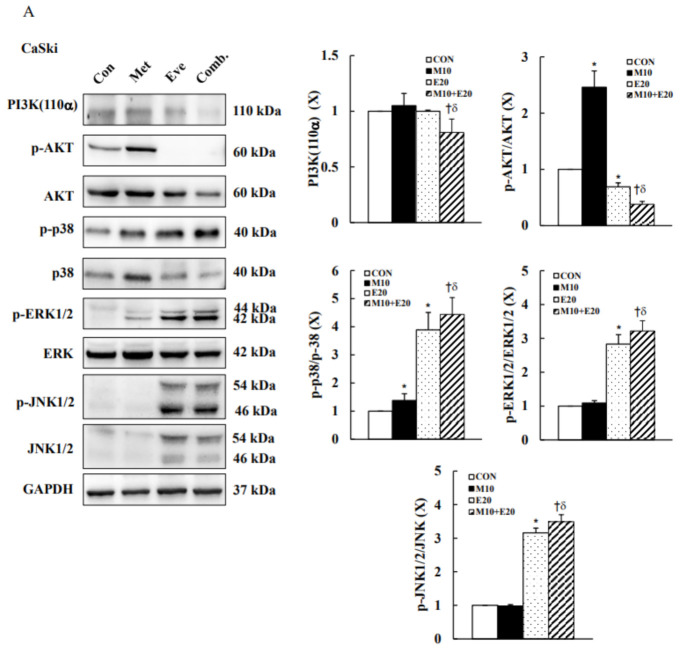

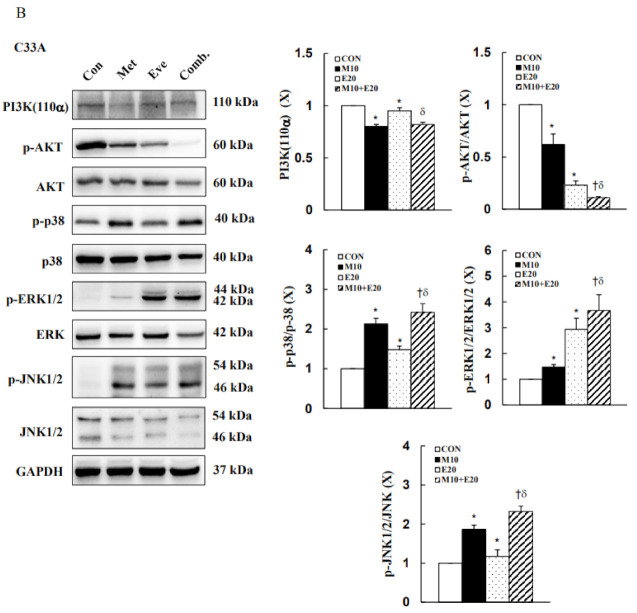
Metformin and everolimus induced apoptosis through the PI3K/AKT and JNK/p38 MAPK signaling pathways in cervical cancer cells. Western blotting was used to detect PIK3CA, p-AKT, AKT, p-p38, p38, p-ERK1/2, ERK1/2, p-JNK1/2, and JNK1/2 levels in (**A**) CaSki and (**B**) C33A cells treated with metformin (10 mM) with or without everolimus (20 μM) for 48 h. GAPDH served as the loading control. Quantitative results are presented in the bottom plot. Values represent the mean ± SD from three replicates. *, † and δ *p* < 0.05 compared with CON, Met 10- or Eve 20-treated groups. CON, 0.1% DMSO; M10, 10 mM metformin; E20, 20 μM everolimus.

To evaluate the effects of metformin and everolimus on cancer-related proteins, the levels of PI3K/AKT and MAPKs were determined using Western blot analyses in CaSki and C33A cells treated with metformin or everolimus alone, or in combination. In CaSki cells, as compared with the control group, the protein levels of PIK3CA, p-ERK1/2, and p-JNK1/2 were unchanged by metformin treatment, while the p-AKT and p-p38 expression levels were significantly increased. Everolimus significantly decreased the expression level of p-AKT and significantly increased the expression levels of p-p38, p-ERK1/2, and p-JNK1/2; the PIK3CA expression level was unchanged (Figure 6A and Figure S3A, *p* < 0.05). In C33A cells, metformin or everolimus monotherapy significantly decreased the expression levels of PIK3CA and p-AKT, and significantly increased those of p-p38, p-ERK1/2, and p-JNK1/2 (Figure 6B and Figure S3B, *p* < 0.05). Moreover, combination treatment with metformin and everolimus significantly decreased the expressions of PIK3CA and p-AKT, and more synergistically increased the p-p38, p-ERK1/2, and p-JNK1/2 expression levels in both CaSki and C33A cells as compared with metformin or everolimus monotherapy. These results suggested that the PI3K/AKT and p38/ERK1/2/JNK1/2 pathways might be involved in metformin- and everolimus-induced cytotoxicity and apoptosis in human cervical cancer cells.

### 3.6. Activations of Caspase-3 and p38 MAPK Are Involved in Metformin- and Everolimus-Induced Apoptosis in Human Cervical Cancer

**Figure 7:**
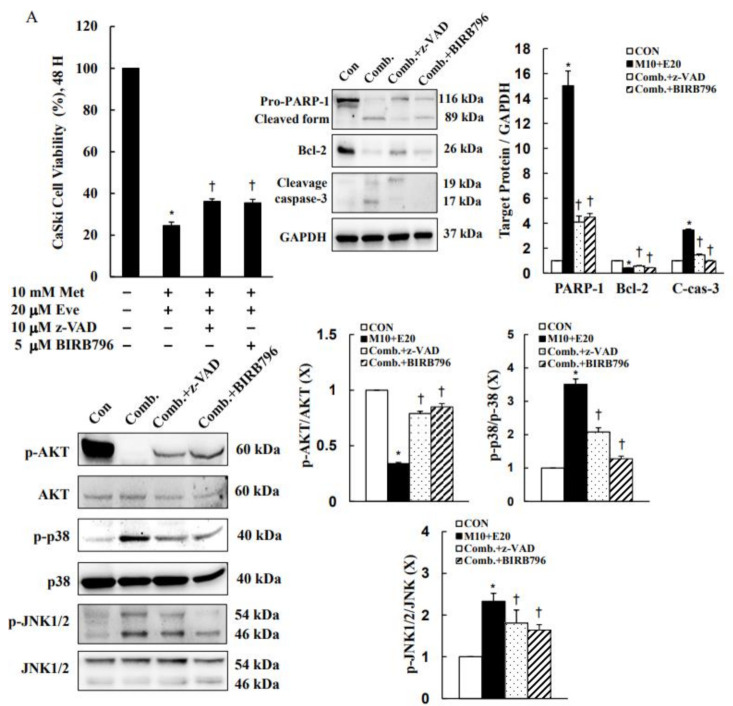

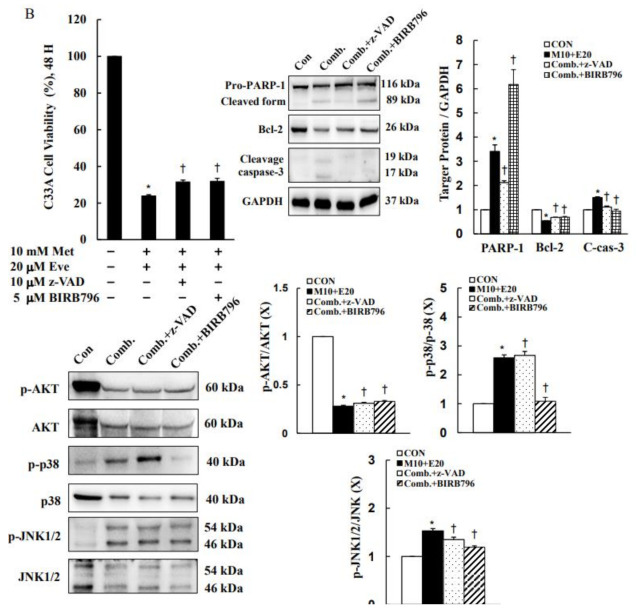
z-VAD-fmk and BIRB796 altered metformin- and everolimus-induced apoptosis-related protein expressions. CaSki and C33A cells were pretreated with or without z-VAD-fmk (10 μM) and BIRB796 (5 μM) for 2 h, then treated with or without metformin (10 mM) and everolimus (20 μM) for 48 h. (**A**) CaSki and (**B**) C33A cell viability was determined using a CCK-8 assay. Protein expression levels of PARP-1, Bcl-2, cleaved caspase-3, p-AKT, AKT, p-p38, p38, p-JNK1/2, and JNK1/2 were assessed using Western blotting. GAPDH served as the loading control. Quantitative results are presented in the bottom plot. Values represent the mean ± SD from three replicates. * and † *p* < 0.05 compared with the CON and Met + Eve-treated groups. CON, 0.1% DMSO; Comb, M10 + E20, 10 μM metformin + 20 μM everolimus; Comb + z-VAD, 10 μM metformin + 20 μM everolimus + 2.5 μM z-VAD-fmk; Comb + BIRB798, 10 μM metformin + 20 μM everolimus + 5 μM BIRB798.

To further validate the role of the caspase-3 and p38 pathways in metformin- and everolimus-induced apoptosis, CaSki and C33A cells were pretreated with z-VAD-fmk (10 μM, a pancaspase inhibitor) or BIRB796 (5.0 μM, a pan-p38 MAPK inhibitor) for 2 h, followed by treatment with metformin and everolimus for 48 h. z-VAD-fmk and BIRB796 significantly ameliorated metformin- and everolimus-induced growth inhibition (24.6 ± 1.7% vs. 36.2 ± 1.2% vs. 35.5 ± 1.7% for CaSki cells; 23.9 ± 0.7% vs. 31.5 ± 1.0% vs. 31.8 ± 1.7% for C33A cells, respectively, *p* < 0.05, Figure 7A,B, Figure S4A,B). Moreover, z-VAD-fmk and BIRB796 both decreased the previously increased levels of cleaved caspase-3, cleaved PARP-1, p38 phosphorylation, and JNK1/2 phosphorylation, and increased Bcl-2 and AKT phosphorylation as compared with metformin with everolimus combined treatment. Similar results were obtained for C33A cells, although the cleaved PARP-1 expression was not reduced after BIRB796 treatment, and p38 phosphorylation was unchanged after z-VAD-fmk treatment. Taken together, these results clearly indicated that metformin combined with everolimus inhibits the growth of cervical cancer cells through a p38-mediated intrinsic apoptosis pathway.

### 3.7. Combined Treatment with Metformin and Everolimus Synergistically Suppresses the Growth of C33A Xenograft Tumors

**Figure 8:**
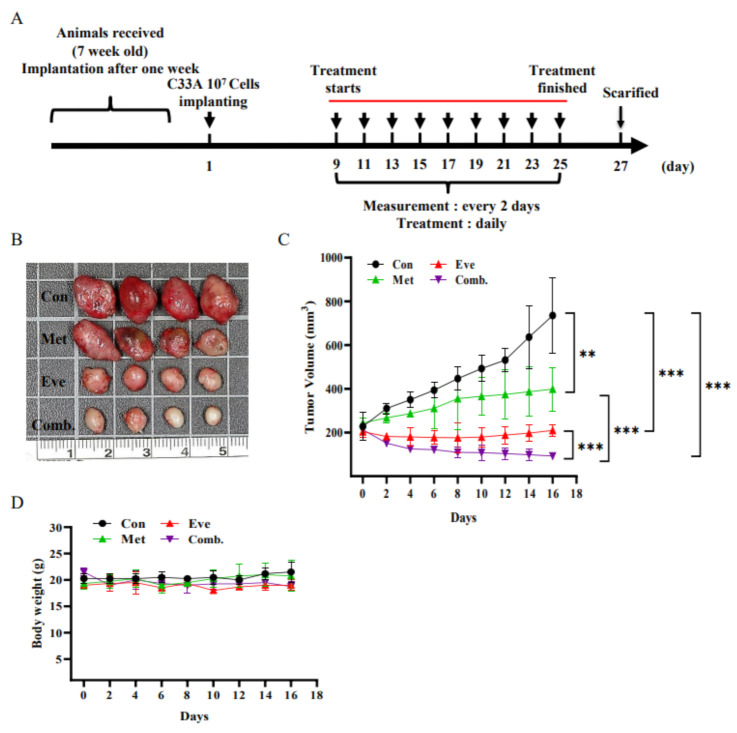

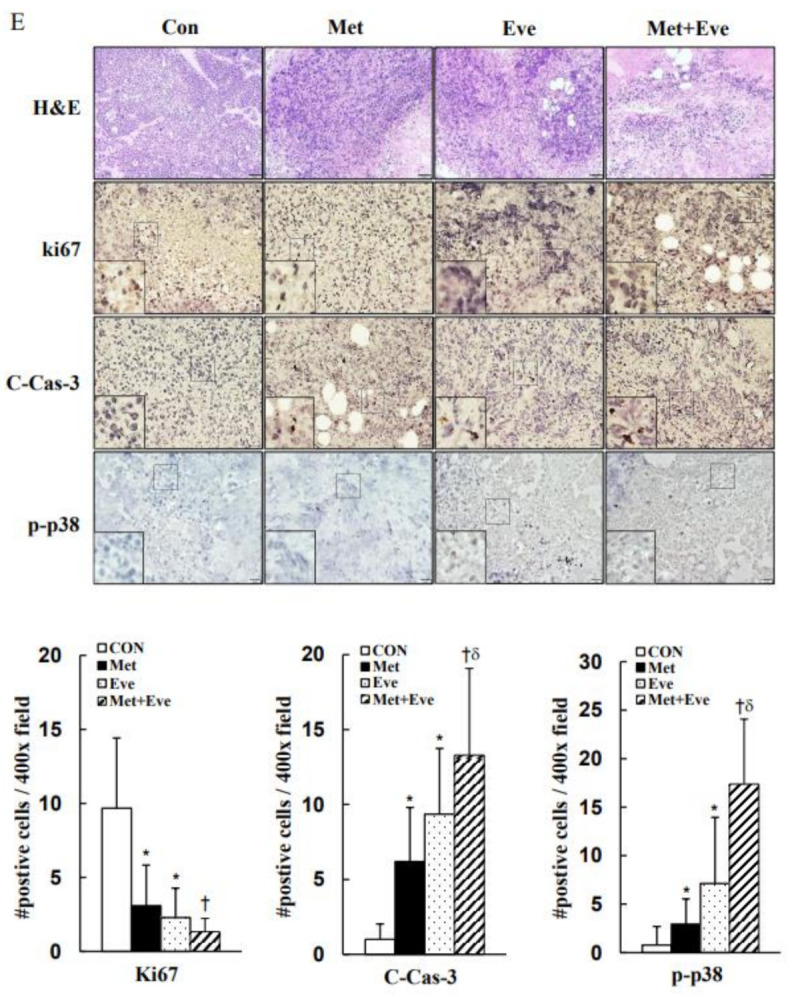
Metformin and everolimus inhibited tumor growth of C33A xenografts in vivo. BALB/c nude mice were subcutaneously injected with C33A cells (1 × 10^7^ cells). When the subcutaneous tumor volume reached ~200 mm^3^, metformin (150 mg/kg), everolimus (5 mg/kg), or vehicle control alone (10% DMSO) was intraperitoneally injected every day. (**A**) Schedule of metformin combined with everolimus treatment, (**B**) representative image of a tumor, (**C**) average tumor volume and (**D**) body weight. *, † and δ *p* < 0.05 compared with CON, Met- or Eve-treated groups. (**E**) Tumor tissue samples were extracted and subjected to HE and immunohistochemical staining to examine the histopathology and expression levels of ki67, cleaved-caspase-3, and p-p38 (shown as brown staining), and quantitative data are presented (H&E, 200×, bar = 50 μm; IHC, 400×, bar = 20 μm). Values represent the mean ± SD (*n* = 4); ** *p* < 0.01, *** *p* < 0.001 compared with CON, control. Met, metformin; Eve, everolimus; Comb., metformin combined with everolimus.

To confirm the antitumor effects of metformin and everolimus in vivo, a C33A xenograft tumor model was generated in BALB/c nude mice. A schematic timeline of this experiment over the five weeks is presented in Figure 8A. The results showed that tumor growth was markedly inhibited in the metformin, everolimus, and metformin plus everolimus groups. Compared with the control group, the tumor xenografts treated with metformin (150 mg/kg/day) and everolimus (5 mg/kg/day) were significantly decreased in size; however, metformin (150 mg/kg/day) plus everolimus (5 mg/kg/day) resulted in a greater significantly decreased tumor size as compared with the other groups (Figure 8B). The tumor volumes of the metformin, everolimus, and metformin plus everolimus groups were 452.8 ± 149.1, 209.3 ± 26.4, and 92.2 ± 15.2 mm^3^, respectively, while that of the control group was 735.7 ± 171.7 mm^3^, with tumor growth inhibition rates of 38.5%, 71.6%, and 87.5%, respectively (Figure 8C, *p* < 0.05). Body weight did not differ significantly among the groups during the experimental period, suggesting that treatment with metformin and everolimus alone or in combination did not induce drug toxicity (Figure 8D). Moreover, metformin and everolimus treatment could significantly prevent histological and morphometric damage to tumor tissue. Similarly, consistent with this study’s in vitro findings, ki67-immunolabeled cells were rarely present in the metformin plus everolimus treatment group as compared with the other groups. In contrast, activated caspase-3 and phospho-P38 immunopositivity were markedly increased after treatment with metformin combined with everolimus as compared with the other groups (Figure 8E). Overall, these results further confirmed that metformin and everolimus combined treatment inhibits cervical cancer tumor growth via targeting the p38 MAPK pathway and activating apoptosis in vivo.

## 4. Discussion

Cervical cancer is the fourth most common cancer in women worldwide, and effective therapies remain limited. Early preclinical data suggested that patients with diabetes have an approximately 1.5- or 2-fold higher risk of some common cancers, such as endometrial (1.61–1.89) and breast (1.56) cancers [44,45]. Chen et al. and Gillani et al. also found that diabetes causes poorer overall survival, poorer recurrence-free survival, and poorer cancer-specific survival in cervical cancer patients [46,47]. Combination chemotherapy decreases the risk of resistance to enhance effectiveness as compared with single-drug therapy for cancer patients; however, multiple drugs may increase the risk of drug interaction and the number of side effects. Currently, only one drug combination is used to treat cervical cancer patients, gemcitabine, and cisplatin (https://www.cancer.gov/about-cancer/treatment/drugs/cervical, accessed on 6 August 2021). Thus, the development of new therapeutic agents and safe and effective treatments is crucial.

Metformin is a widely used drug for the treatment of type II diabetes and has an important anticancer property. Recent studies have focused on combining metformin with commonly used clinical chemotherapeutic drugs to decrease their toxicity and attenuate tumor resistance in various types of cancer [48]. Thus, in this study, we used human cervical cancer cell lines CaSki (HPV-16, -18) and C33A (HPV-negative) in addition to a mouse xenograft tumor model to assess whether metformin combined with everolimus has a greater effect against cancer than that of everolimus alone, and investigated the underlying mechanisms of this combined treatment. Our study results indicated that metformin combined with everolimus therapy could significantly inhibit cell growth (as shown by reduced cell viability and PCNA protein level) and promote cell apoptosis (as evidenced by upregulation of Bax, cleaved-PARP-1, and cleaved caspase-3 protein levels) in both human cervical cancer cell lines (Figure 1, Figure 2 and Figure 5); this combined treatment also inhibited xenograft animal tumor growth to produce markedly synergistic anticancer effects in a mouse model (Figure 8). These results were consistent with those previously reported by Liu et al. and Wang et al., who identified that the combined use of metformin and everolimus synergistically augmented anticancer activity in the treatment of breast cancer [35,49]. In addition, the results of the present study indicated that CaSki cells were more sensitive to metformin and everolimus treatment, which significantly induced cell apoptosis, than C33A cells (Figure 2B), which was consistent with our previous study results [38]. Hsieh et al. and Xiao et al. showed that metformin could reduce p53, activate LKB1 and AMPK, and inhibit mTOR via the inhibition of HPV, resulting in induced apoptotic HeLa and MS571 cell death, whereas cervical cancer cells both HPV- and LKB1-negative were less sensitive to metformin [50,51].

As previously reported, metformin induced cytotoxicity by promoting cell-cycle arrest in the G0/G1 (SW480, K7M2, and MG63 cells) [52,53,54] or G2/M (HCT116 p53^−/−^, 143B, and U20S cells) phase [54,55], and everolimus was involved in G0/G1-phase (MCF-7, BT474, and K562 cells) [56,57] and G2/M-phase arrest (Rb MCF-7 and gemcitabine-resistant MIAPaCa cells) [58,59]. In the present study, metformin combined with everolimus also caused G0/G1-phase cell-cycle arrest in CaSki and C33A cells, in addition to a higher percentage of cells in the sub-G1 phase in CaSki cells, but not in C33A cells (Figure 3). These differences observed in the cell-cycle phase arrest effects of metformin and everolimus treatment may be caused by the cancer cell type specificity in a time- or dose-dependent manner. In 1924, Otto Warburg showed that oxidative phosphorylation (OXPHOS) is crucial for ATP production and tumor progression, which is impaired in cancer cells [60]. In addition, Payen et al. revealed that mitochondrial ROS exhibit both tumor-suppressing and -promoting roles, and high levels of mtROS induce tissue damage and cause cell death [61]. Our results showed that metformin combined with everolimus significantly enhanced Complex I, III, V subunit levels to preserve mitochondrial ETC function, increase mtROS production, and prompt cancer cell apoptosis (Figure 4). These results were consistent with those reported by Li et al. and Imhoff et al., who revealed that metformin-induced cell apoptosis and autophagy were caused by the induction of high levels of ROS-induced mtROS [62,63]. In fact, our previous study showed that metformin alone or combined with pitavastatin upregulated the Complex I expression to improve the efficacy of OXPHOS in pancreatic cancer cells [39]. Several studies have shown that metformin could directly inhibit Complex I efficacy and attenuate ROS formation to reduce tumorigenesis [20,64,65]. Therefore, the differing inhibition of Complex I efficacy could be attributed to different metformin concentrations in the matrix, thereby affecting the mitochondrial membrane potential. In 2000, El-Mir et al. and Owen et al. reported that, in some cases, treatment with 10 mmol/L metformin did not inhibit the Complex I efficacy, and quite a high concentration (up to 10 mmol/L) and a long incubation period were required [66,67]. However, several studies also revealed that Complex I activity was extremely high when the metformin dose was in the range of 20–80 mmol/L [68,69]; thus, the dose- and time-dependent manner of metformin may modulate the extent of Complex I activation. In fact, other studies showed that high-dose metformin exerts a direct anticancer effect, which requires adequate metformin accumulation in neoplastic tissue to inhibit mTOR and fatty acid synthesis via APMK activation and induce cellular energetic stress and energetic crisis, leading to tumor cell death [70,71]. Zakikhani et al. revealed that treatment with 5~20 mM metformin more effectively reduced cell proliferation of breast (MCF-7), ovary (SKOV3 and OVACR-3), and prostate (PC-3) cancer cells through up-regulation of AMPK and down-regulation of mTOR and p70S6K [40].

The PI3K/AKT signaling pathway is a target for cancer treatment owing to its hyperactivation in tumor cells, and the protein expression levels of PI3K and p-AKT are inhibited by metformin or everolimus in some common cancers, including cervical cancer [56,72,73,74]. Meanwhile, the growth of cervical cancer cells was found to be inhibited by a combination of metformin and nelfinavir; combining everolimus with paclitaxel treatment was observed to inhibit the PI3K/AKT/mTOR pathway expression, which is consistent with our study findings that everolimus combined with metformin therapy inhibited PI3K and p-AKT protein expressions in CaSki and C33A cells (Figure 6). Furthermore, p-p38, p-ERK, and p-JNK activations were induced in CaSki and C33A cells after metformin and/or everolimus treatment. Caspase inhibitor z-VAD-fmk and p38 MAPK inhibitor BIRB796 significantly reversed CaSki and C33A cell growth and alleviated p38 MAPK activation in cells treated with metformin combined with everolimus (Figure 7), which can be explained by the fact that p38, ERK, and JNK promote human cervical cancer cell death by regulating metformin- and/or everolimus-induced cell apoptosis. These findings were consistent with those of previous reports demonstrating that metformin induced apoptosis or autophagy through activating the ROS/JNK cascade in human osteosarcoma, and through activating the JNK/p38 MAPK pathway in lung cancer cells [62,75]. Everolimus also reportedly resulted in a mTORC1-MAPK feedback loop to activate the MAPK pathway, regulating cell growth in human prostate and gastric cancers [76,77]. Tseng et al., however, revealed that metformin combined with paclitaxel therapy decreased p38 MAPK signaling activation in human NSCLC cells [78]. These inconsistencies explain why the combination of metformin with chemotherapeutic drugs acts via differing molecular mechanisms and may be dependent on cell type and stimuli.

## 5. Conclusions

**Figure 9:**
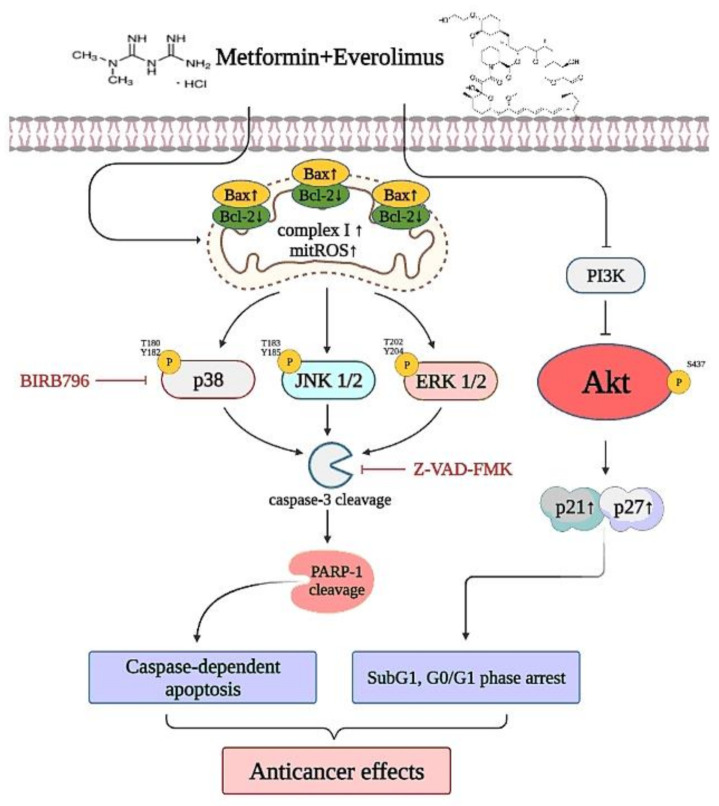
Anticancer molecular mechanism of metformin combined with everolimus in cervical cancer treatment. Metformin sensitizes everolimus, which mainly modulates MAPKs (p38, ERK, and JNK) upregulation and PI3K/AKT downregulation, activating or inactivating various signaling targets, such as Bax, Bcl2, caspase-3, PARP-1, Complex I, mtROS, p21, and p27. The activation and inactivation of these signaling pathways induced subG1- and G0/G1-phase arrest and caspase-dependent apoptosis, which increase the anticancer effects on cervical cancer.

This study was the first to evaluate the antitumor effects of metformin combined with everolimus on human cervical cancer in vitro and in vivo, and to examine the potential molecular mechanisms. Metformin combined with everolimus induced caspase-dependent apoptotic cell death in human cervical cancer cells via mtROS/MAPK signaling, and metformin combined with everolimus induced sub-G1- and G0/G1-phase arrest via PI3K/AKT signaling (Figure 9). Furthermore, in vivo study showed that metformin combined with everolimus indirectly inhibited xenograft tumor growth, and the cytotoxic effect of metformin combined with everolimus was minimal or nonexistent. Collectively, these results revealed that metformin combined with everolimus provides a novel therapeutic strategy for patients with cervical cancer.

## Figures and Tables

**Table 1:** Clinical studies of agents in combination with metformin.

Disease	Combined Drug(s)	No. of Patients	Finding	Ref.
Endometrial cancer	Megestrol acetate compared with megestrol acetate alone	150	Significant in 102 atypical endometrial hyperplasia (AEH) patients (39.6 versus 20.4%, OR 2.56, *p* = 0.04).	[24]
Refractory colorectal cancer	lrinotecan	41	In the multivariate analysis, disease control at 12 weeks impacted overall survival HR 0.21, *p* = 0.001.	[25]
Pancreatic cancer	Gemcitabine, erlotinib	121	Overall survival at 6 months was 56.7% (95% CI 44.1–69.2) in the metformin group and 63.9% (51.9–75.9) in the placebo group (*p* = 0.41).	[26]
Epithelial ovarian cancer	Chemotherapy versus chemotherapy alone	44	The disease-free survival (DFS) and progression-free survival (PFS) of patients with metformin use versus without metformin use was 29 versus 26 months (*p* = 0.61) and 23 versus 21 months (*p* = 0.68), respectively.	[27]
Metastatic pancreatic cancer	(Ir)relevance of metformin treatment	60	Six-months progression-free survival (PFS-6) was 52% (95% CI 33–69) in the control group and 42% (24–59) in the metformin group (*p* = 0.61).	[28]
Prostate cancer	metformin plus standard of care or standard of care alone	124	Castration-resistant prostate cancer-free survival was improved with metformin (29 months (95% CI 25–33) versus 20 months (16–24); *p* = 0.01).	[29]
Breast cancer	Chemotherapy versus chemotherapy alone	122	Median Progression-free survival (PFS) was 9.4 months (95% CI 7.8–10.4) in with metformin intake group and 9.9 (7.4–11.5) in without metformin intake group (*p* = 0.651).	[30]

## Data Availability

The datasets generated during and/or analysed during the current study are available from the corresponding author on reasonable request.

## Supplementary Materials

The following are available online at https://www.mdpi.com/article/10.3390/cancers13184612/s1, Figure S1: Metformin and everolimus enhanced OXPHOS expression; Figure S2: Metformin and everolimus activates mitochondrial and caspase-mediated pathways; Figure S3: Metformin and everolimus regulated PI3K/AKT and JNK/p38 MAPK signaling pathways; Figure S4: Caspase-3 and p38 MAPK involves metformin- and everolimus-induced apoptosis.
