# Supplementary material for: Metformin Potentiates the Anticancer Effect of Everolimus on Cervical Cancer In Vitro and In Vivo
Source: Cancers (Basel). 2021 Sep 14;13(18):4612. doi: 10.3390/cancers13184612 (PMC8468269; doi:10.3390/cancers13184612)
Supplement: Supplementary file 1 [file cancers-13-04612-s001.zip › cancers-1372821-supplementary.pdf]

Supplementary Materials: Metformin Potentiates the Anti-cancer Effect of Everolimus on Cervical Cancer In Vitro and In Vivo

Ya-Hui Chen, Jyun-Xue Wu, Shun-Fa Yang, Mei-Ling Chen, Tze-Ho Chen and Yi-Hsuan Hsiao

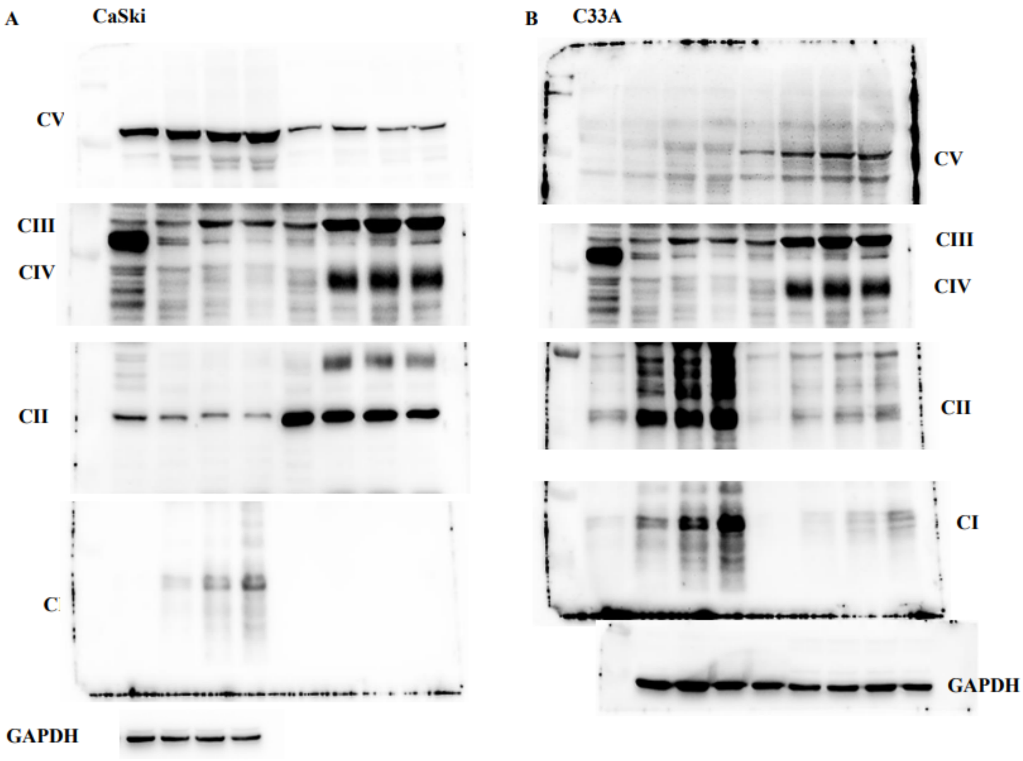

Figure S1. Metformin and everolimus enhanced OXPHOS expression.

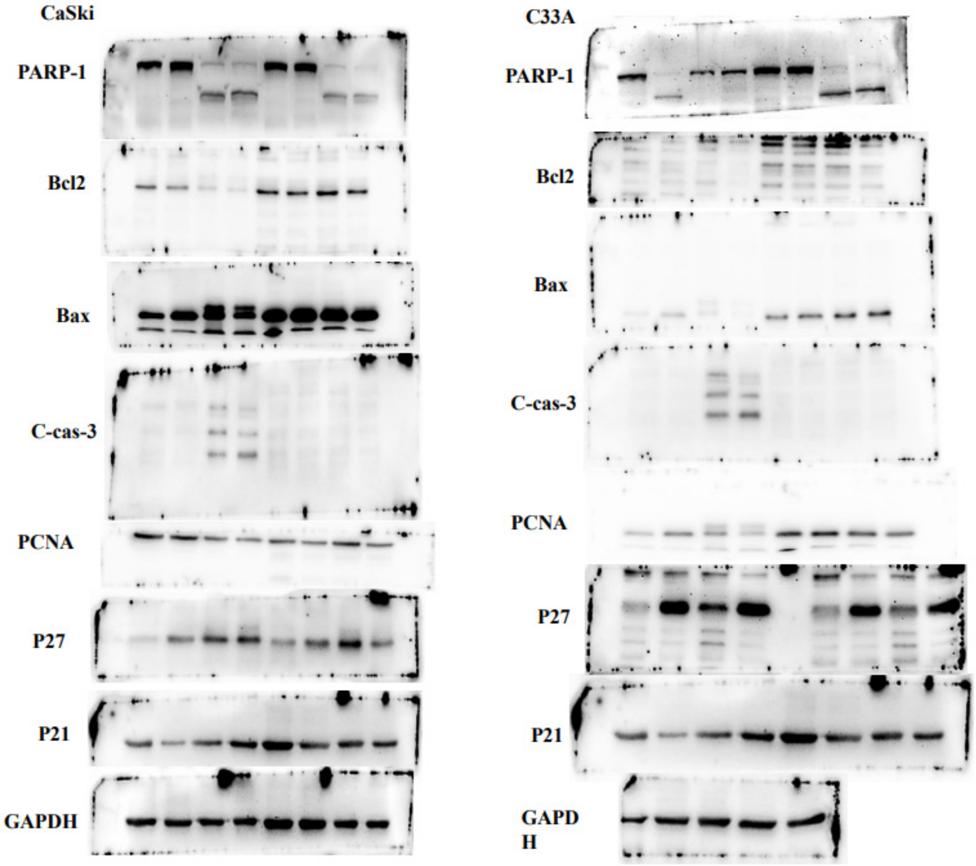

Figure S2. Metformin and everolimus activates mitochondrial and caspase-mediated pathways.

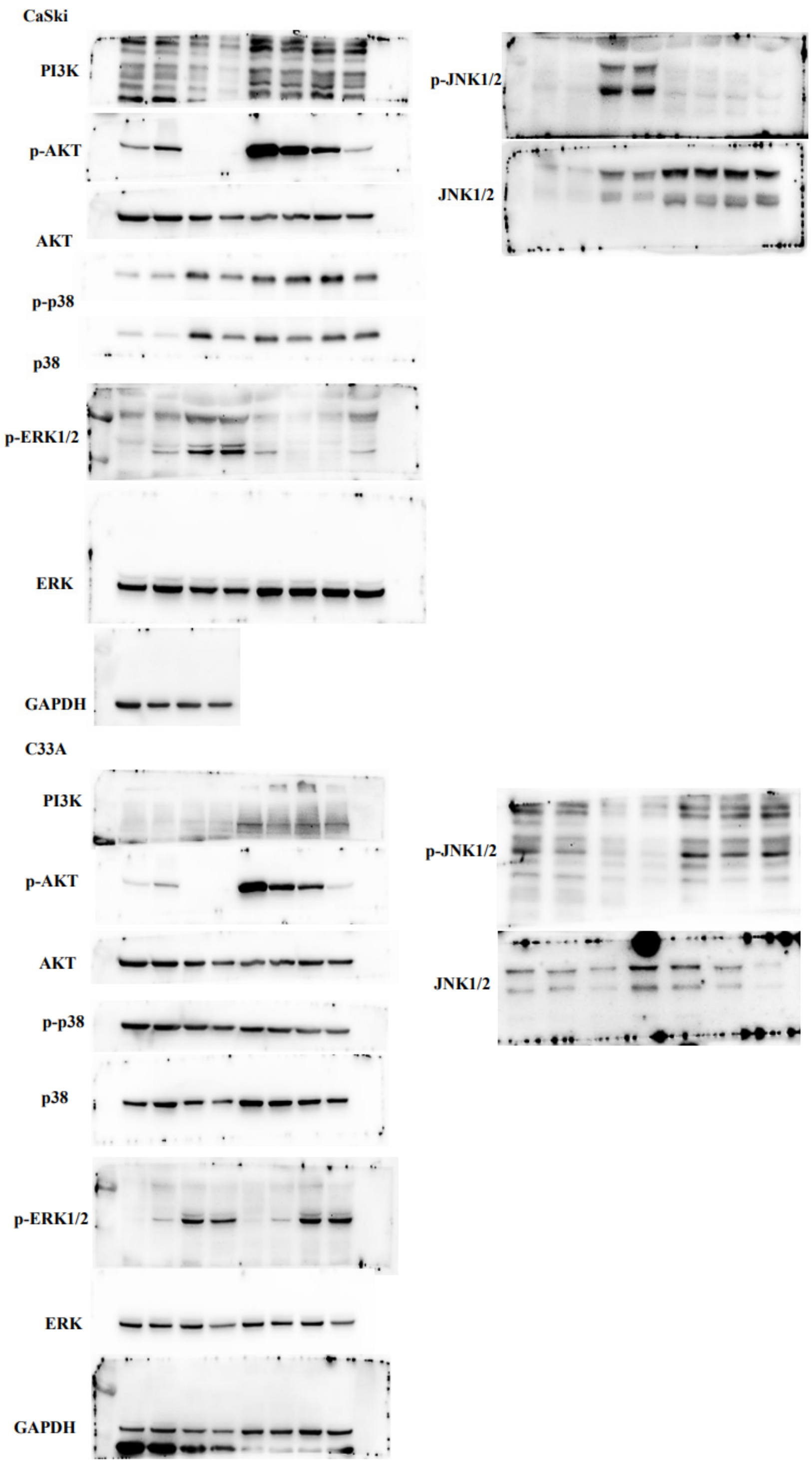

Figure S3. Metformin and everolimus regulated PI3K/AKT and JNK/p38 MAPK signaling pathways.

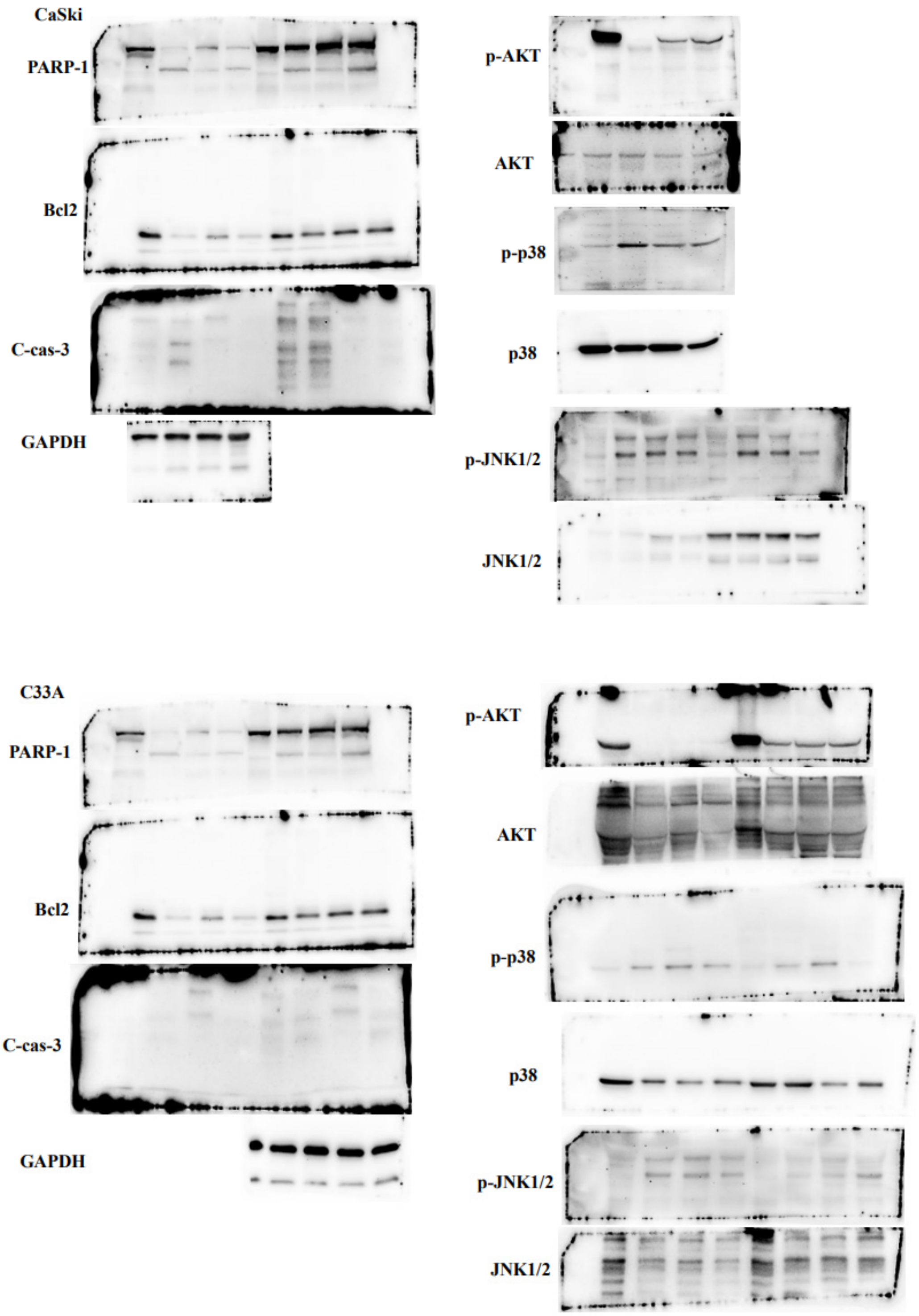

Figure S4. Caspase-3 and p38 MAPK involves metformin- and everolimus-induced apoptosis.
